# Prognostic value of serum levels of multiple adhesion factors in patients with sepsis-induced acute kidney injury

**DOI:** 10.1007/s11255-022-03394-z

**Published:** 2022-11-04

**Authors:** Yan Li, Qingsheng Huang, Mingxing Fang, Mengyao Liu, Jianying Guo, Zhiyong Wang

**Affiliations:** Department of Critical Care Medicine, Third Hospital of HeBei Medical University, The 139rd of ZiQiang Road, ShiJiaZhuang, 050051 China

**Keywords:** Sepsis, Acute kidney injury, Adhesion factors, E-selectin, L-selectin, P-selectin, ICAM-1, VCAM-1

## Abstract

**Background:**

Acute kidney injury (AKI) is common in patients with sepsis and septic shock. Urine output and serum creatinine (SCr) levels are the criteria for diagnosing AKI. However, the application of these levels in the diagnosis of AKI has limitations.

**Objective:**

To detect the expression of various adhesion factors in different stages of AKI as defined by Kidney Disease: Improving Global Outcomes (KDIGO) and to analyse their relationship with the prognosis of patients with sepsis-induced AKI (S-AKI).

**Methods:**

Adult patients with sepsis who were admitted to the hospital between June 2019 and May 2020 were included. Of 90 adult patients with sepsis, 58 had S-AKI. Sixty-seven subjects without sepsis were used as controls. Enzyme-linked immunosorbent assay kits were used to measure E-selectin (CD62E), L-selectin (CD62L), P-selectin, intercellular adhesion molecule 1 (ICAM-1) and vascular cell adhesion molecule 1 (VCAM-1), and their relationship with the prognosis of patients with S-AKI patients was analysed. Receiver operating characteristic curves were used to analyse the predictive value of different adhesion factors on renal resistance index and renal function recovery. Multivariate logistic regression analysis was used to identify factors associated with renal recovery.

**Results:**

The expression of CD62L was significantly higher in S-AKI patients than in non-AKI patients with sepsis. Compared with the non-AKI group, Acute Physiology and Chronic Health Evaluation II and Sequential Organ Failure Assessment scores were significantly higher in the AKI group than in the non-AKI group (*P* < 0.05). Mean blood pressure, SCr levels and procalcitonin levels were higher in the AKI group than in the non-AKI group (*P* < 0.05 for all). The CD62L levels decreased with increasing S-AKI stage. The CD62E levels were highest in S-AKI stage 2, and the VCAM-1 levels were highest in S-AKI stage 3. All patients with S-AKI were followed up with for 28 days. The results found that VCAM-1 was the best predictor of renal recovery in patients with S-AKI.

**Conclusion:**

CD62L is an indicator of S-AKI stage1, and CD62E is an indicator of S-AKI stage 2. In addition, VCAM-I demonstrated satisfactory performance in predicting early recovery of renal function in patients with S-AKI.

## Introduction

Acute kidney injury (AKI) associated with sepsis (S-AKI) is frequent in patients with sepsis and septic shock. According to earlier research, 57% of critically ill patients in intensive care units (ICUs) have acute kidney injury. Approximately 50% of these patients develop AKI secondary to sepsis, and AKI occurs in 50–70% of patients with sepsis [1–4]. Most current recommendations, including those from Kidney Disease: Improving Global Outcomes (KDIGO), regard serum creatinine (SCr) levels and urine output as diagnostic indicators of AKI. Even though SCr is easily measured, there are limitations to its use in diagnosing AKI. For example, renal hypoperfusion may result in a higher SCr without significant renal parenchymal damage. Moreover, reliance on increased SCr can delay early diagnosis and treatment of AKI, as approximately 50% of patients with kidney injury do not have an increased SCr [5–8]. Therefore, it is necessary to identify other sensitive and specific biomarkers for the early diagnosis of AKI.

Previous studies have recommended using several early biomarkers for the diagnosis of AKI based on their varied activities and connections with damage to different regions of the nephron unit. Ischaemic damage raises the levels of kidney injury molecule-1 (KIM-1), neutrophil gelatinase-associated lipocalin (NGAL), monocyte chemoattractant protein-1 and cysteine-rich angiogenic inducer-61. In contrast, hypoxic damage increases the level of liver fatty acid-binding protein. Glomerular injury and a lower estimated glomerular filtration rate led to increased levels of SCr, cystatin C, and NGAL. Proximal tubule injury leads to higher levels of KIM-1, N-acetyl-β-D-glucosaminidase, insulin-like growth factor-binding protein-7 and tissue inhibitor of metalloproteinases-2. In contrast, distal convoluted tubule damage leads to increased levels of NGAL [9].

Sepsis affects the glomeruli and renal tubules by producing renal haemodynamic abnormalities, activating immune cells, releasing inflammatory chemicals and downregulating endogenous hormones [10]. Many studies have explored renal haemodynamics and biomarkers of tubular injury, but research on the immune system’s interaction with sepsis and AKI is scarce and controversial. According to reports, AKI has anti-inflammatory and inhibitory effects on neutrophil granulocytes [11]. There is additional evidence that renal tubular cells and other significant epithelial cells are associated with adhesion factor release in tissue injury. Renal tubular epithelial cells might express vascular cell adhesion molecules and cell adhesion molecules, resulting in the recruitment and activation of T lymphocytes, subsequently activating the cluster of differentiation-40 and harming epithelial cells [12].

Given the above reasons, this study aims to determine the expression of various adhesion factors in the different phases of AKI defined by KDIGO and examine their relationship with the prognosis of patients diagnosed with S-AKI.

## Patients and methods

### Participants and groups

In this prospective study, adult patients with sepsis who were admitted between June 2019 and May 2020 and had a 28-day follow-up appointment were included. Sepsis was defined as per the Sepsis-3 criteria [13]^.^ The inclusion criteria were as follows: (1) sequential organ failure assessment (SOFA) score ≥ 2 points in diagnosed or suspected infection; (2) blood lactic acid level above 2.0 mmol/L. The exclusion criteria were as follows: (1) potential chronic kidney disease or patients on maintenance haemodialysis; (2) age less than 18 years; (3) pregnancy; (4) renal vascular abnormalities or previous renal surgery, such as kidney transplant; (5) ICU stay less than three days; or (6) patients unwilling to participate.

The control group included patients without sepsis. The inclusion criteria were: (1) willingness to participate in the entire study. (2) age > 18 years; and (3) no abnormality on physical examination. The exclusion criteria were as follows: (1) history of a genetic type of nephropathy. (2) Previous history of AKI; and (3) pregnancy.

The Ethics Committee of the Third Hospital of Hebei Medical University approved this study (approval number: KE-2019-20-1, KE-2019-19-1), and each participant signed informed consent before participation.

Acute kidney injury was diagnosed according to the 2012 KDIGO criteria [14]: an increase in SCr of more than 26.5 μmoL/L within 48 h, a 50% or greater rise of SCr from the baseline or a urine output of less than 0.5 mL/kg/h for more than six h. Patients with S-AKI were also classified by stage (S-AKI-1, S-AKI-2 and S-AKI-3) and were followed up with for 28 days. According to international consensus criteria [15] and a previous study [16], renal recovery was defined as a restoration of SCr to within 150% of baseline level without requiring renal replacement therapy (RRT). Non-recovery was defined as the need for RRT or no restoration of the SCr level at 28 days after admission.

### Intervention measures

Following the Surviving Sepsis Campaign guidelines for achieving resuscitation goals [17], septic patients were given conventional treatment, which included fluid resuscitation and vasoactive drugs to maintain a mean arterial pressure (MAP) of at least 60 mmHg, a superior vena caval blood oxygen saturation of at least 70% and the lowest possible central venous pressure using restrictive fluid management once shock was corrected.

### Data collection

Data collected from all subjects at admission were age, gender, underlying disease(s), site(s) of infection, Acute Physiology and Chronic Health Evaluation II (APACHE II) and SOFA scores within 24 h of entering the ICU, systolic blood pressure, diastolic blood pressure and MAP. The following parameters were recorded: laboratory results, including white blood cell (WBC) count, platelet count, lactic acid, procalcitonin (PCT), SCr, blood urea nitrogen, duration of ICU stay and 28-day mortality.

### Testing of adhesion factors

Blood and urine samples were collected when patients entered the ICU and after 24 h and 72 h. Samples were centrifuged for 15 min, and the soluble and supernatant fractions were collected and stored at  – 80 °C before testing. Blood samples were sent to the laboratory for routine blood and biochemical examination. Enzyme-linked immunosorbent assay (ELISA) kits (Arigo Biolaboratories, China) were used to measure the serum levels of E-selectin (CD62E), L-selectin (CD62L), P-selectin (CD62P), intercellular adhesion molecule 1 (ICAM-1) and vascular cell adhesion molecule 1 (VCAM-1).

### Statistical analysis

All data were analysed using the IBM SPSS Statistics for Windows, Version 25.0 (Armonk, NY). Continuous variables were expressed as means ± standard deviation ($\overline{X }\pm S$) and were compared using either a *t*-test or a one-way analysis of variance, as appropriate. Categorical variables were expressed as numbers and percentages, and comparisons between groups were performed using the chi-square (χ^2^) test or Fisher’s exact test. Receiver operating characteristic (ROC) curves were used to analyse the predictive value of different adhesion factors on the renal resistive index and recovery. Multiple logistic regression analysis was used to identify factors associated with renal recovery. A *P*-value of less than 0.05 was considered statistically significant.

## Results

### Baseline characteristics of sepsis patients with and without AKI

In total, 90 septic patients and 67 patients without sepsis were enrolled (Table 1). Among the septic patients, 32 had pulmonary infections, 16 had abdominal cavity infections, 6 had blood infections and 28 had soft tissue infections. Seventy-eight of the septic patients had AKI (59%). The APACHE II and SOFA scores were significantly higher in the AKI group as compared with the non-AKI group (*P* < 0.001 for both). The mean blood pressure (MBP), SCr level and PCT levels were also higher in the AKI group as compared with the non-AKI group (*P* < 0.001 for all). There were no significant differences in WBC counts between the two groups, but the platelet count was significantly lower in the AKI group (*P* < 0.001). In addition, patients in the AKI group had a significantly longer length of hospital stay and higher 28-day mortality (*P* < 0.05 for both).

**Table 1:** Comparisons of baseline characteristics of individuals in the healthy control and sepsis groups, and of sepsis patients in the AKI and non-AKI groups

Variable	Control	Sepsis	*P*-value	SAKI	Non-SAKI	*P*-value
*N*	67	90		52	38	
Age, years	41.0 ± 15.7	67.0 ± 21.3	< 0.001	71.5 ± 23.5	60.9 ± 16.3	0.013
Female, *N* (%)	25 (37.3)	33 (36.7)	0.934	15 (28.8)	18 (47.4)	0.072
SBP, mmHg	126.9 ± 16.6	108.8 ± 19.3	< 0.001	103.3 ± 19.1	116.3 ± 17.1	0.001
DBP, mmHg	66.3 ± 6.7	59.0 ± 13.9	< 0.001	56.1 ± 14.6	63.1 ± 11.9	0.016
MBP, mmHg	86.5 ± 7.0	75.6 ± 14.9	< 0.001	71.8 ± 15.4	80.9 ± 12.4	0.004
24-h APACHE II	6.1 ± 7.5	21.1 ± 6.7	< 0.001	24.2 ± 5.7	16.8 ± 5.7	< 0.001
24-h SOFA	2.0 ± 3.1	10.1 ± 4.6	< 0.001	12.5 ± 3.4	6.7 ± 3.8	< 0.001
*Infection site*						
Intra-abdominal		16 (17.8)		7 (13.5)	9 (23.7)	
Respiratory tract		32 (35.6)		28 (53.8)	4 (10.5)	
Soft tissue		28 (31.1)		7 (13.5)	21 (55.3)	
Blood		4 (4.4)		4 (7.7)	0 (0.0)	
WBCs (10^9^/L)	10.2 ± 7.4	15.4 ± 11.2	0.001	15.7 ± 13.0	14.9 ± 8.4	0.763
Platelets (10^9^/L)	217.7 ± 116.6	134.5 ± 119.6	< 0.001	96.1 ± 83.8	187.0 ± 140.8	0.001
SCr, μmol/L	75.2 ± 33.0	158.4 ± 151.6	< 0.001	219.8 ± 175.3	74.4 ± 18.6	< 0.001
PCT, ng/mL	1.5 ± 2.1	19.1 ± 30.2	< 0.001	29.8 ± 35.5	4.5 ± 8.7	< 0.001
Length of ICU stay, days	13.5 ± 34.2	34.4 ± 54.7	0.004	51.2 ± 66.9	11.4 ± 9.7	< 0.001
28-day mortality, *N* (%)	3 (4.5)	27 (30.0)	< 0.001	22 (42.3)	5 (13.2)	0.003

The Sepsis includes SAKI and Non-SAKI. (*SAKI* Sepsis-associated acute kidney injury, *Non-SAKI* Non Sepsis-associated acute kidney injury.) *SBP* systolic blood pressure, *DBP* diastolic blood pressure, *MBP* mean blood pressure, *APACHE II* Acute Physiology and Chronic Health Evaluation II, *SOFA* sequential organ failure assessment, *WBC* white blood cell, *SCr*: serum creatinine, *PCT* procalcitonin

### Biomarkers and prognosis of patients with different stages of AKI

According to the KDIGO criteria [18], 27 patients had AKI-1, 13 had AKI-2 and 18 had AKI-3 (Table 2). A comparison of the three AKI groups indicated significant statistical differences in age (*P* = 0.013) but not gender (*P* = 0.432). However, there were other significant differences among these three groups. In particular, the AKI-2 group had a higher APACHE II score and a lower MBP than the AKI-1 and AKI-3 groups. These three groups also had significant differences in PCT levels, with the highest level in the AKI-2 group. A comparison of patients with sepsis with and without AKI indicated that the AKI patients had greater expression of CD62L, CD62E and VCAM-1. Notably, the level of CD62L decreased as the AKI stage increased. The level of CD62E was highest in the AKI-2 group, and the level of VCAM-1 was highest in the AKI-3 group, followed by the AKI-2 and AKI-1 groups but without a statistical difference (*P* > 0.05).

**Table 2:** Clinical factors, biomarkers, and prognosis of the different groups

Variable	Total	Non-AKI	AKI stage			*P*-value
			AKI-1	AKI-2	AKI-3	
*N*	157	99	27	13	18	
Age, yrs	55.9 ± 23.0	47.8 ± 18.9	74.7 ± 17.8	77.4 ± 18.6	56.9 ± 27.8	0.013
Female, *N* (%)	58 (36.9)	43 (43.4)	5 (18.5)	4 (30.8)	6 (33.3)	0.432
SBP, mmHg	116.5 ± 20.2	123.2 ± 18.0	107.8 ± 17.3	89.3 ± 11.3	112.7 ± 19.7	0.001
DBP, mmHg	62.1 ± 11.9	64.8 ± 9.1	55.9 ± 12.4	49.4 ± 8.3	66.3 ± 17.2	0.003
MBP, mmHg	80.3 ± 13.3	84.2 ± 9.9	73.2 ± 13.3	62.7 ± 8.8	81.8 ± 17.6	0.002
24-h APACHE II	14.7 ± 10.3	9.6 ± 8.7	22.0 ± 7.0	28.2 ± 5.2	21.9 ± 3.3	0.003
24-h SOFA	6.6 ± 5.7	3.4 ± 3.8	10.9 ± 4.5	13.5 ± 1.9	12.9 ± 3.2	0.067
WBCs (10^9^/L)	13.2 ± 10.1	12.1 ± 8.2	18.8 ± 13.2	9.3 ± 7.8	13.3 ± 12.9	0.062
Platelets (10^9^/L)	170.0 ± 125.0	215.4 ± 124.3	65.7 ± 62.5	117.2 ± 78.9	115.1 ± 96.8	0.058
SCr, μmol/L	122.9 ± 123.6	70.0 ± 19.3	126.3 ± 52.4	136.2 ± 73.4	400.3 ± 178.8	< 0.001
PCT, ng/mL	15.0 ± 27.5	3.5 ± 7.2	16.3 ± 28.6	30.0 ± 33.1	41.6 ± 40.1	0.053
CD62P, pg/ml	4155.0 ± 399.1	4195.4 ± 349.9	3958.3 ± 569.7	4220.9 ± 289.7	4347.5 ± 200.9	0.469
CD62L, ng/mL	46.5 ± 19.1	39.3 ± 14.7	67.9 ± 24.3	54.1 ± 9.8	48.4 ± 8.0	0.002
CD62E, pg/mL	2328.1 ± 1549.0	2162.2 ± 1591.3	2332.9 ± 1515.1	3044.5 ± 1430.0	2716.2 ± 1324.1	0.168
VCAM-1, pg/mL	1146.5 ± 660.0	1099.9 ± 704.0	1147.0 ± 629.3	1271.3 ± 454.8	1312.0 ± 577.9	0.616
ICAM-1, ng/mL	15.8 ± 7.2	14.9 ± 7.3	16.9 ± 6.7	19.7 ± 6.0	16.5 ± 6.8	0.374
Renal artery resistance index	0.7 ± 0.1	0.6 ± 0.0	0.7 ± 0.0	0.7 ± 0.0	0.7 ± 0.0	0.008
Length of ICU stay, days	25.5 ± 48.1	12.9 ± 28.7	71.3 ± 85.7	35.1 ± 32.1	18.6 ± 11.0	0.018
28-day mortality, *N* (%)	30 (19.1)	5 (5.1)	12 (44.4)	8 (61.5)	5 (27.8)	0.170

*SBP* systolic blood pressure, *DBP* diastolic blood pressure, *MBP* mean blood pressure, *APACHE II* Acute Physiology and Chronic Health Evaluation II, *SOFA* sequential organ failure assessment, *WBC* white blood cell, *SCr* serum creatinine, *PCT* procalcitonin, *CD62E* E-selectin, *CD62L* L-selectin, *CD62P* P-selectin, *VCAM-1* vascular cell adhesion molecule 1, *ICAM-1* intercellular adhesion molecule 1

CD62L: AKI-1vs AKI-0, *P* < 0.05; AKI-1 vs AKI-2, *P* > 0.05; AKI-1 vs AKI-3, *P* < 0.05; AKI-2 vs AKI-3, *P* > 0.05

CD62E: AKI-1vs AKI-0, *P* > 0.05; AKI-0 vs AKI-2, *P* > 0.05; AKI-0vs AKI-3, *P* > 0.05; AKI-2 vs AKI-3, *P* > 0.05; AKI-2 vs AKI-1, *P* > 0.05

VCAM-1: AKI-1vs AKI-0, *P* > 0.05; AKI-0 vs AKI-3, *P* > 0.05; AKI-2vs AKI-3, *P* > 0.05; AKI-2 vs AKI-1, *P* > 0.05

ICAM-1: AKI-1vs AKI-0, *P* > 0.05; AKI-2vs AKI-3, *P* > 0.05; AKI-0 vs AKI-3, *P* > 0.05; AKI-2 vs AKI-3, *P* > 0.05; AKI-2 vs AKI-1, *P* > 0.05

### Univariate analysis of factors associated with renal recovery in patients with S-AKI

All 52 patients with S-AKI were followed up with for 28 days. Fifteen of these patients (28.8%) had recovery of renal function (see Table 3). Univariate analysis identified lower APACHE II and SOFA scores and WBC counts in the renal recovery group, with *P* < 0.05. The levels of CD62P, CD62L, CD62E, ICAM-1 and VCAM-1 were all lower in the renal recovery group, but the difference was not statistically significant. The renal recovery group’s 28-day mortality rate was also lower (*P* < 0.05).

**Table 3:** Univariate analysis of factors associated with renal recovery in patients with S-AKI

Variable	Total	Recovery	Non-recovery	*P*-value
*N* (%)	52 (100.0)	15 (28.8)	37 (71.2)	
Age, years	71.5 ± 23.5	60.4 ± 31.7	76.0 ± 18.0	0.090
Female, *N* (%)	15 (28.8)	4 (26.7)	9 (32.1)	1.000
SBP, mmHg	103.3 ± 19.1	105.5 ± 25.3	103.4 ± 16.6	0.649
DBP, mmHg	56.1 ± 14.6	56.5 ± 16.4	56.7 ± 14.4	0.887
MBP, mmHg	71.8 ± 15.4	72.7 ± 18.2	72.4 ± 14.4	0.893
24-h APACHE II	24.2 ± 5.7	20.5 ± 4.1	25.7 ± 5.7	0.002
24-h SOFA	12.5 ± 3.4	10.3 ± 3.6	13.4 ± 3.0	0.003
WBCs (10^9^/L)	15.7 ± 13.0	6.3 ± 4.4	19.4 ± 13.5	< 0.001
Platelets (10^9^/L)	96.1 ± 83.8	87.2 ± 41.5	99.8 ± 96.1	0.512
SCr, μmol/L	219.8 ± 175.3	173.3 ± 173.9	235.5 ± 177.6	0.121
PCT, ng/mL	29.8 ± 355	17.7 ± 26.3	34.7 ± 37.7	0.073
CD62P, pg/mL	4075.8 ± 457.1	3998.7 ± 496.3	4127.2 ± 469.4	0.689
CD62L, ng/mL	57.6 ± 20.0	55.0 ± 6.0	58.7 ± 23.1	0.403
CD62E, pg/mL	2647.5 ± 1454.2	2710.3 ± 1391.5	2622.1 ± 1496.8	0.845
VCAM-1, pg/mL	1230.8 ± 602.9	1048.2 ± 440.2	1304.8 ± 648.1	0.167
ICAM-1, ng/mL	17.6 ± 6.6	16.1 ± 7.4	18.1 ± 6.3	0.320
Length of ICU stay, days	51.2 ± 66.9	75.7 ± 62.7	41.2 ± 66.8	0.092

*SBP* systolic blood pressure; *DBP* diastolic blood pressure; *MBP* mean blood pressure; *APACHE II* Acute Physiology and Chronic Health Evaluation II, *SOFA* sequential organ failure assessment, *WBC* white blood cell, *SCr* serum creatinine, *PCT* procalcitonin, *CD62E* E-selectin, *CD62L* L-selectin, *CD62P* P-selectin, *VCAM-1* vascular cell adhesion molecule 1, *ICAM-1* intercellular adhesion molecule 1

### Receiver operating characteristic analysis of patients with S-AKI

**Fig. 1:**
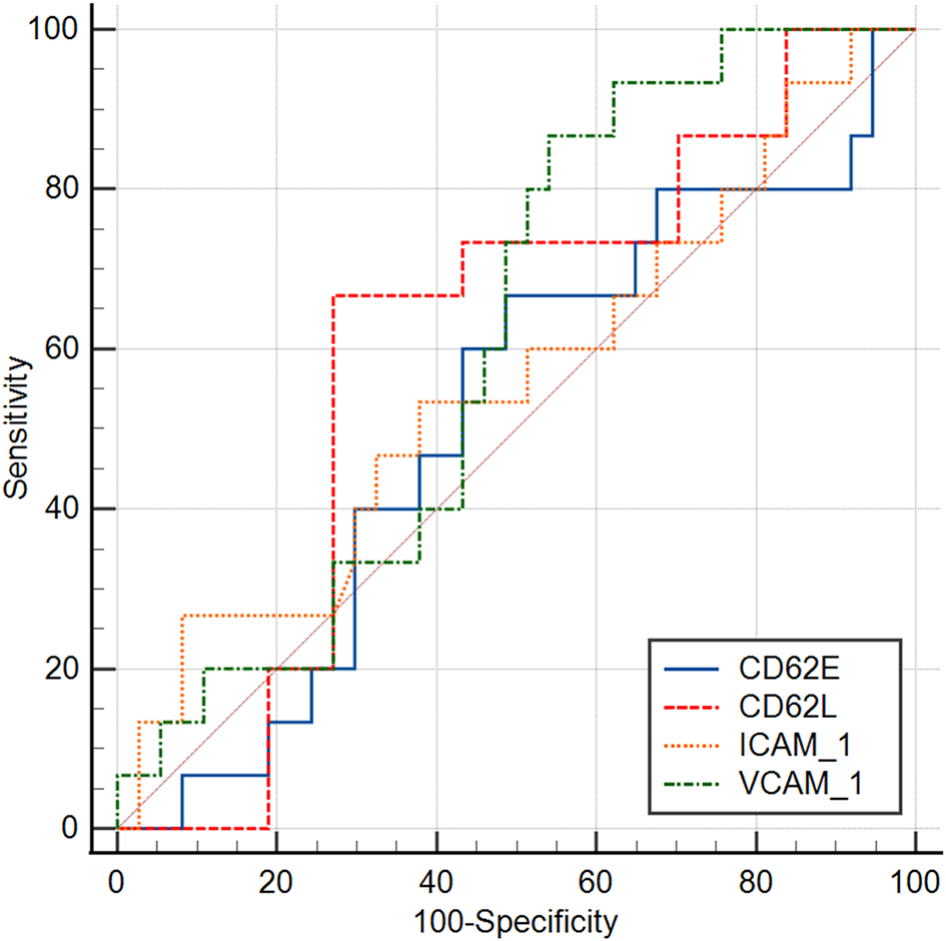
Receiver-operating curves of adhesion factors for predicting the recovery of patients with septic acute kidney injury in 28-day follow-up

A ROC analysis was performed for each of the four blood markers, using the optimal cut-off values based on Youden’s J statistic, to assess their performance in predicting renal recovery in patients with S-AKI (Table 4). The results indicated that VCAM-1 provided the best diagnostic performance (area under the curve [AUC] = 0.61, sensitivity = 0.867 and specificity = 0.460) and CD62E provided the worst diagnostic performance (AUC = 0.52, sensitivity = 0.667 and specificity = 0.514) (Fig. 1).

**Table 4:** Receiver operating characteristic analysis of the relationship of different adhesion molecules with renal recovery in patients with S-AKI

Variable (optimal cut-off)	AUC (95% CI)	Younden’s J	Sensitivity	Specificity
CD62L (55.13 ng/ml)	0.60 (0.46, 0.74)	0.396	0.667	0.730
CD62E (2785.06 pg/mL)	0.52 (0.37, 0.66)	0.180	0.667	0.514
VCAM-1 (1303.43 pg/mL)	0.61 (0.47, 0.75)	0.326	0.867	0.460
ICAM-1 (9.14 ng/ml)	0.56 (0.41, 0.70)	0.186	0.267	0.919

*CD62E* E-selectin, *CD62L* L-selectin, *CD62P* P-selectin, *VCAM-1* vascular cell adhesion molecule 1, *ICAM-1* intercellular adhesion molecule 1

### Multiple logistic analysis of factors associated with death in patients with S-AKI

Multiple logistic regression analysis showed that the levels of CD62L, CD62E, VCAM-1 and ICAM-1 were not significantly associated with 28-day mortality (*P* > 0.05 for all) (Table 5).

**Table 5:** Multiple logistic analysis of factors associated with death from S-AKI

Variable	Regression coefficient (b)	Standard error (Sb)	Wald’s χ^2^	*P*-value	OR (95% CI)
CD62L	– 0.004	0.015	0.070	0.792	0.996 (0.968, 1.025)
CD62E	0.000	0.000	2.084	0.149	1.000 (0.999, 1.000)
VCAM-1	0.001	0.001	0.585	0.444	1.001 (0.999, 1.002)
ICAM-1	0.055	0.060	0.841	0.359	1.056 (0.939, 1.188)

*CD62E* E-selectin, *CD62L* L-selectin, *CD62P* P-selectin, *VCAM-1* vascular cell adhesion molecule 1, *ICAM-1* intercellular adhesion molecule 1

## Discussion

This study explored the expression of various adhesion factors in different stages of AKI as defined by KDIGO and evaluated their diagnostic value for predicting the prognosis of patients with S-AKI. The main findings can be summarised as follows: (1) Compared with the non-AKI group, MBP, SCr levels, PCT levels, APACHE II scores and SOFA scores were significantly higher in the AKI group than in the non-AKI group. (2) The expression of CD62L was significantly higher in patients with S-AKI than in patients with sepsis without AKI. (3) Among the multiple adhesion factors, VCAM-1 provided the best predictive value for the 28-day renal recovery in patients with S-AKI. These results suggest that some adhesion factors change significantly during the recovery of renal function and, therefore, might be useful tools for risk stratification and clinical decision-making.

White cells [19] and inflammatory markers [20] were raised in all stages of AKI, including onset, progress and recovery. Due to the functions of CD62E, CD62L, CD62P and integrins, neutrophil granulocytes adhere to the inflammatory site of the kidney [21, 22]. Inflammation can eradicate infections, but it also increases tissue and organ damage. Inflammatory mediators simultaneously stimulate epithelial cells and upregulate the expression of adhesion factors such as CD62E, which accelerate the recruitment of white blood cells to the kidney. The continuance of this process, the increase in vascular permeability and the activation of adhesive factors further promote the inflammatory reaction [23–25]. Therefore, inflammation and leukocyte fundraising are at the core of sepsis development, and these processes play a role in a positive feedback cycle that exacerbates severity. In this study, the three adhesion-related factors (CD62L, CD62E and VCAM-1) of patients with S-AKI were higher than those with sepsis. In addition, the ROC analysis showed that CD62L, CD62E and VCAM-1 levels were related to renal function recovery of patients with S-AKI.

The study found that the expression of CD62L, VCAM-1 and CD62E was greater in patients with S-AKI when compared with patients with sepsis alone. Moreover, the CD62L level appeared to decline as AKI progressed, in that its expression was highest in the S-AKI-1 group, followed by the S-AKI-2 and S-AKI-3 groups. The CD62E level appeared to at first increase and then decrease as AKI progressed, in that its expression was highest in the S-AKI-2 group, followed by the S-AKI-3 and S-AKI-1 groups. The level of VCAM-1 was highest in the S-AKI-3 group, followed by the S-AKI-2 and S-AKI-1 groups. The level of ICAM-1 was highest in the S-AKI-2 group, followed by the S-AKI-1 and S-AKI-2 groups. These results suggest that CD62L are indicators of S-AKI-1, CD62E is an indicator of AKI-2 and ICAM-1 is an indicator of AKI-2. Previous AKI research has shown that the peak of neutrophil recruitment in the kidneys occurs approximately 24 h after injury [26]. This finding is consistent with the results in this study in that the levels of the adhesion factor CD62L were higher in patients with S-AKI-1 than in those with S-AKI-2 and S-AKI-3. The results of other studies have shown that cytokine levels declined after neutrophil migration, and cytokine release promoted leukocyte recruitment and increased vascular permeability [27]. Thus, the levels of several adhesion factors increase soon after AKI onset and then decline as AKI progresses.

Endothelial cells are crucial in the control of coagulation. Under normal circumstances, the endothelium controls blood volume, electrolyte balance and blood coagulation, preventing microcirculatory diseases such as thrombotic microangiopathy and disseminated intravascular coagulation [28]. Due to their ability to be detected even before conventionally recognised signs, endothelial biomarkers may be used as indicators of endothelial damage [29]. This study demonstrated that among the various adhesion factors, VCAM-1 had the best diagnostic value for predicting the recovery of patients with S-AKI. Additionally, VCAM-1 has been investigated as a diagnostic and prognostic agent for illness since it is only detectable after cytokine activation of endothelial cells [29, 30]. In the study by Mota et al. [31], serum VCAM-1 in the AKI group was considerably higher than in the control and non-AKI groups, suggesting that endothelial cells in the AKI group had been more activated and injured. When used to predict AKI within 24 h of a two-headed snake bite, VCAM-1 performed very well, which is consistent with the findings of this study; VCAM-1 also demonstrated remarkable accuracy when identifying AKI within 24 h of moderate envenomation after a two-headed snake bite. Thus, VCAM-1 might provide valuable information for risk stratification in practice.

The follow-up of S-AKI patients found that the CD62P, CD62L, CD62E, ICAM-1 and VCAM-1 levels in patients who did not recover were low, but the difference was statistically significant only for VCAM-1. The 28-day mortality rate in the renal recovery group was significantly lower than in the non-resilient group. Therefore, these results indicate that low levels of adhesion factors indicate early recovery of renal function in patients with S-AKI and that the recovery of renal function increases survival [32]. These results are like the results of other studies, and patients with early kidney recovery have the same long-term survival as patients with sepsis but no AKI. Therefore, early diagnosis and treatment of AKI and early kidney recovery seem essential to improving the prognosis of patients with S-AKI.

The damage to vascular endothelial cells and the activation, adhesion and infiltration of leukocytes play crucial roles in the progression of sepsis [33]. Both ICAM-1 and VCAM-1 are adhesion molecules of the immunoglobulin family, expressed by endothelial cells. Their primary function is to induce leukocytes to adhere to endothelial cells firmly, thus promoting an increase in circulating levels of ICAM-1 and VCAM-1 [34]. This study’s ROC analysis showed that VCAM-1 demonstrated high sensitivity and specificity in predicting an early renal recovery in patients with S-AKI and that CD62E had high sensitivity but low specificity in predicting an early renal recovery in these patients. Several recent studies have attempted to identify factors associated with early renal recovery in patients with AKI, but the results have been inconclusive. For example, Fiorentino et al. showed that the APACHE II score and the baseline SCr were independent predictors of recovery of renal function in patients with S-AKI [32]. The Biological Markers of Recovery for the Kidney study, BioMaRK, showed that a baseline clinical model that included age, MBP, mechanical ventilation and bilirubin could predict renal recovery and mortality similar to plasma biomarkers. However, the clinical model also considered plasma IL-8 to have a higher predictive value for renal recovery and mortality with AUCs of 0.76 and 0.78, respectively [35]. The prediction model of this study examined several additional biomarkers: CD62L, CD62E, CD62P and VCAM-1, and found that CD62L and VCAM-1 demonstrated a high value for predicting a renal recovery in patients with S-AKI. This study thus suggests a new approach for predicting the prognosis of patients with S-AKI, although studies with larger samples are needed for confirmation.

The multiple logistic regression analysis showed that adhesion factors and PCT showed no significant correlation with 28-day mortality in patients with S-AKI. In contrast, other research [34] has reported that the ICAM-1 and VCAM-1 levels correlated significantly with organ dysfunction and mortality in patients with sepsis. This difference may be due to the small sample size in this study, a low number of deaths or differences in the study populations. Extensive prospective studies are needed to confirm the correlation between serum levels of adhesion factors and deaths in patients with S-AKI.

This study has several limitations. This is a single-centre, prospective study with a relatively small sample size. The results may be vulnerable to selection bias, raising concerns about generalisability. In addition, ELISA was used to determine the level of adhesive factors in plasma. However, the level of these factors in the kidney and other tissues could not be determined. Further, some of the baseline data in this study were not comparable. Therefore, further large and multicentre studies are needed to reveal the underlying mechanism of these adhesion factors in the pathogenesis and recovery of patients with S-AKI.

## Conclusion

CD62L is an indicator of S-AKI-1 and CD62E is an indicator of S-AKI-2. In addition, VCAM-I showed satisfactory performance in predicting early recovery of renal function in patients with S-AKI.

## Abbreviations

S-AKI: Sepsis-induced acute kidney injury
ICU: Intensive care unit
CD62E: E-selectin
CD62L: L-selectin
CD62P: P-selectin
ICAM-1: Intercellular adhesion molecule 1
VCAM-1: Vascular cell adhesion molecule 1
KDIGO: Kidney disease: improving global outcomes
SCr: Serum creatinine
VCAMs: Vascular cell adhesion molecules
ICAMs: Intercellular adhesion molecules
RRT: Renal replacement therapy
MAP: Mean arterial pressure
APACHE II: Acute physiology and chronic health evaluation II
WBC: White blood cell
PCT: Procalcitonin
MBP: Mean blood pressure
ELISA: Enzyme-linked immunosorbent assay
SOFA: Sepsis-related organ failure assessment
ROC: Receiver operating characteristic
